# Effect of iron on the expression of *sirR *and *sitABC *in biofilm-associated *Staphylococcus epidermidis*

**DOI:** 10.1186/1471-2180-6-103

**Published:** 2006-12-19

**Authors:** Caroline Massonet, Valerie Pintens, Rita Merckx, Jozef Anné, Elke Lammertyn, Johan Van Eldere

**Affiliations:** 1 Lab medical microbiology, Department Medical Diagnostic Sciences, KULeuven, U.Z.Gasthuisberg, Herestraat 49 CDG8th floor, B-3000, Leuven, Belgium; 2 Lab Bacteriology, Department Microbiology and Immunology, KULeuven, Rega institute, Minderbroedersstraat 10, B-3000, Leuven, Belgium

## Abstract

**Background:**

Different gene expression patterns correlate with the altered phenotype in biofilm-associated bacteria. Iron and iron-linked genes are thought to play a key-role in biofilm formation. The expression of Fe-linked genes (s*irR*, *sitABC *operon) in *Staphylococcus epidermidis*, was compared in planktonic versus sessile bacteria *in vitro *and *in vivo *in a subcutaneous foreign body rat model.

**Results:**

*In vitro *in a Fe-limited environment, the planktonic form of *S. epidermidis *produces siderophores and grows slower than in Fe-rich environment. The expression of s*irR *in planktonic bacteria, *in vitro*, was not different in medium without Fe or with 1 μM FeCl_3_. High Fe concentrations (25 μM FeCl_3_) increased expression of *sirR *transiently during the early phase of incubation. Expression of *sitC in vitro*, in planktonic bacteria, was inversely correlated with *sirR *expression in medium with 25 μM FeCl_3_: *sitC *expression decreased for the first 3 hours followed by an up regulation.

In sessile bacteria *in vitro*, *sirR *expression was high and independent of the Fe concentration. The expression of *sitC *was not inversely correlated to *sirR *expression.

*In vivo*, expression levels of *sirR *and of *sitABC *were high during the initial phase after implantation and, after a transient decrease, remained stable over a period of two weeks.

**Conclusion:**

Our data suggest that the expression of *sirR *and the regulatory effect of *sirR *on the *sitABC *operon are different in planktonic and sessile bacteria.

## Background

The free ionic iron (Fe) concentration in the human body is kept at a very low level to limit bacterial growth. This is achieved through Fe-carrier proteins like transferrin and lactoferrin that bind ferric Fe (Fe^3+^) with a high affinity. Pathogenic bacteria however have developed powerful mechanisms that are capable of chelating Fe at very low concentrations.

Coagulase negative staphylococci (CoNS) and in particular *S. epidermidis *are the most common cause of foreign body associated infections (FBI) such as infections of prosthetic valves, pacemakers, orthopedic prostheses and cerebrovascular shunts. A typical aspect of these FBI are the so-called biofilms consisting of multilayered clusters of CoNS that are attached to the hard surface and embedded in a slime layer [1,2].

Limited data are available regarding Fe-scavenging mechanisms in *Staphylococcus epidermidis*. A cell surface Fe-receptor to obtain Fe from the receptor-bound transferrin has been reported [3] and another mechanism involves the production of siderophores [4,5]. These low molecular mass ligands chelate free Fe and were also shown to release Fe from transferrin due to their higher affinity for Fe [6-8]. The cell surface receptor and siderophores need to be complemented by another mechanism that mediates the uptake of Fe into the bacterial cell. Cockayne *et al*. [9] and Hill *et al*. [5] have suggested that the *sitABC *operon contains genes that could be involved in Fe uptake. The *sitABC *operon gene products (SitA, SitB and SitC) together constitute an ABC-transporter with homology to the multifunctional ABC operons involved in adherence and genetic competence in streptococci [5,10,11]. The products of s*itA, sitB *and *sitC *also show homology to the products of the *mntABC *operon (Manganese (Mn) transporter) in *S. aureus *[12]. *SitA *encodes an ATP binding protein and *sitB *codes for a membrane protein. The third open reading frame (s*itC*) encodes a lipoprotein that is anchored in the cytoplasmic membrane [9] and could act as a ferric siderophore receptor or could be involved in shuttling Fe from the transferrin receptor across the cell wall to the cytoplasmic membrane prior to uptake into the cell [9,13]. The genes of the *sitABC *operon have a single common promoter region, and the expression of *sitC *is considered representative for the expression of the complete operon [5,9]. SitC is a 32 kDa cell membrane protein that is very immunogenic and Fe-repressible. *In vitro *studies by Cockayne *et al*. [9] and Hill *et al*. [5] showed that *sitC *is expressed in a Fe-limited environment but not in a Fe-rich environment. SitC shows homology to a number of bacterial adhesins like, EfaA, ScaA and FimA in *Enterococcus faecalis*, *Streptococcus gordonii *and *Streptococcus parasanguis*, respectively. Because SitC remains tethered to the cytoplasmic membrane by its lipid tail, it can function in siderophore dependent and independent Fe acquisition pathways. Furthermore, it is unlikely that SitC plays a specific role in the adhesion of *S. epidermidis *because of its limited exposure to the surface [14]. According to Modun *et al*. [15] and Cockayne *et al*. [9], cell envelope proteins like SitC could be important virulence factors and could be toxic to mammalian cells. It is interesting that release of significant quantities of these staphylococcal membrane lipoproteins into culture supernatant under Fe-restricted growth conditions *in vivo *and *in vitro *was observed [9].

Located adjacent to this *sitABC *operon is *sirR*. SirR is a metallo-dependent repressor that has homology with DtxR and DtxR homologues like MntR in *S. aureus*, especially since the metal coordination sites are preserved [5,6,12]. According to Hill *et al*. [5] a putative operator site of SirR could be found in the sequence of the promoter region of the *sitABC *operon is This operator site is a sir-box, a region of dyad symmetry that overlaps the transcriptional start of *sitABC *in the promoter region of the operon. It has also been reported by Hill *et al*. [5] that if the genes of the ABC transporter are regulated through SirR, they are transcriptionally regulated through SirR in a Fe- or Mn-concentration dependent manner. Intracellular ferrous Fe (Fe^2+^) could attach to the metal coordination sites leading to conformational changes in the Fe^2+^-SirR complex that facilitate binding to a sir-box [5]. Several sir-boxes are found in the genome of *S. epidermidis *and SirR might, similar to DtxR and Fur-homologues, be a pleiotropic regulator of gene expression in this bacterium [5]. According to Hill *et al*. [5], expression of *sirR *in planktonic (in suspension) bacteria is not affected by the Fe concentration. It remains to be established whether other regulatory mechanisms can affect *sirR *expression. Additionally, it is not yet clear whether expression of *sirR *is different in sessile (biofilm-associated) bacteria due to the specific metabolic conditions in biofilm or due to biofilm formation itself.

The expression of genes of the *sitABC *operon in planktonic bacteria on the other hand is affected by the Fe and Mn concentration, as has been shown in *in vitro *studies [5,9]. In these studies, no expression of *sitC *was observed after 18 hrs of growth in a medium with Fe or in a Fe-free medium with Mn [5,9]. Earlier studies also indicated that the *sitABC *operon was expressed *in vivo *[15] and *in vitro *[16] in an Fe-depleted medium in planktonic bacteria. Studies on the *mntABC *operon and its regulator *mntR *gene in *S. aureus *found that the transcription of the operon was affected more by the Fe concentration than by the Mn concentration [12]. According to Horsburgh *et al*. [17] Mn plays a role in the protection of a number of bacteria, like *Bacillus subtilis *and *S. aureus *against oxidative stress.

Bacteria in a biofilm display significant phenotypical and genotypical changes [18]. Because of this, the concentration range of an essential growth factor like Fe may be different for sessile and planktonic cell growth [18]. *In vivo *and *in vitro*, sessile bacteria respond to a more narrow Fe concentration range than planktonic bacteria [18].

In this study we examined the expression of the *sitABC *operon and *sirR *during biofilm formation by *S. epidermidis*, *in vitro *in the presence and the absence of Fe and *in vivo *in a FBI rat model.

## Results

### Effect of Fe on growth kinetics and siderophore production

Bacteria from an overnight culture of *S. epidermidis *in RPMI 1640 depleted of Fe, (fRPMI) were resuspended in 100 ml fRPMI with 1 μM FeCl_3 _(=fRPMI-Fe1) and without Fe at a density equivalent to an OD_600 _of approximately 0.005.

**Figure 1:**
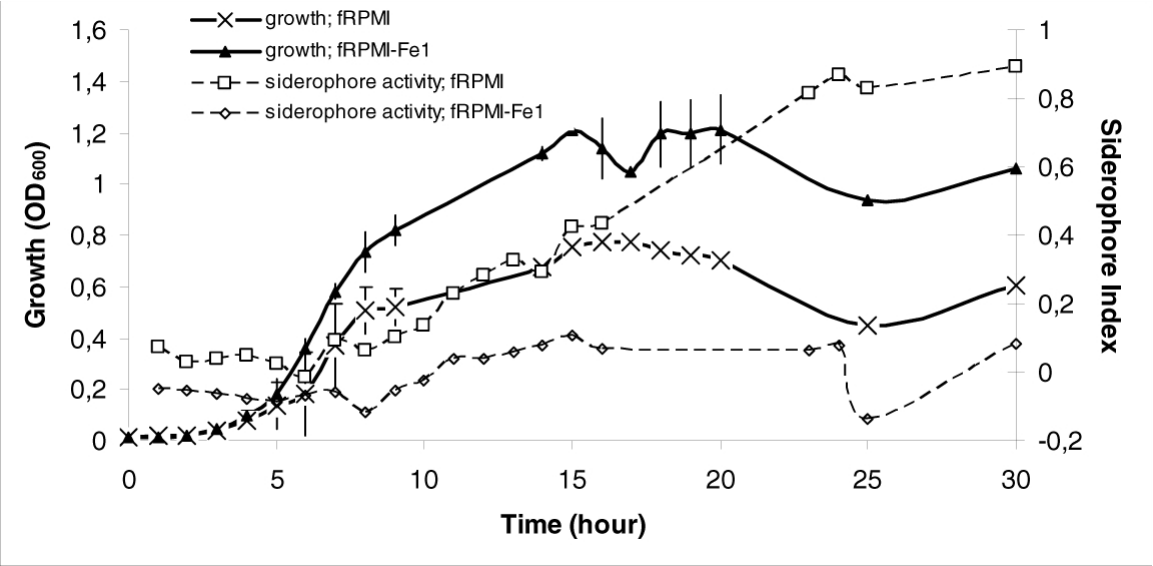
Growth kinetics and siderophore production of *S. epidermidis *(planktonic growth) in fRPMI and fRPMI-Fe1. Bacteria were overnight pre-incubated in fRPMI and incubated in fRPMI or in fRPMI-Fe1. The right y-axis, the dotted line with empty diamonds (fRPMI-Fe1) and the dotted line with empty squares (fRPMI) represent siderophore activity, measured at 630 nm. The left y-axis, the full line with filled triangles (fRPMI-Fe1) and the full line with crosses (fRPMI) represent the growth curve, given as OD_600_. The x-axis contains the time in hours. The line that links the data points helps to clarify the results at each time point measured. The error bars represent standard deviations. At each time point 15 samples from three independent cultures (five samples from each culture) were assessed.

With inocula of 10^6 ^cells/ml, (OD_600 _= 0.005) the lag phase was shorter and the growth yield was higher in fRPMI-Fe1 than in fRPMI. The exponential growth of *S. epidermidis *in fRPMI was slower than in fRPMI-Fe1 (Fig. 1). Stationary phase was attained after approximately 18 hrs, followed by a slight decrease in bacterial density after 25 hrs and 30 hrs in both media. With inocula of 10^7 ^cells/ml, the lag phase also was shorter in fRPMI-Fe1 than in fRPMI. The stationary phase was reached sooner for both media in comparison to the lower inoculum and the difference in final bacterial yield (after 30 hrs) between both media was smaller for 10^7 ^cells/ml than for 10^6 ^cells/ml (data not shown).

The presence of siderophores reflected the presence or absence of Fe in the medium. In Fe-rich medium (1 μM FeCl_3_) the siderophore production remained at a constant low level over a time period of 30 hrs. In the absence of Fe, the siderophore production increased and a maximal siderophore production was achieved after 30 hrs (Fig. 1).

### Effect of pre-incubation and incubation conditions on the expression of *sirR *and *sitC *in planktonic bacteria *in vitro*

**Figure 2:**
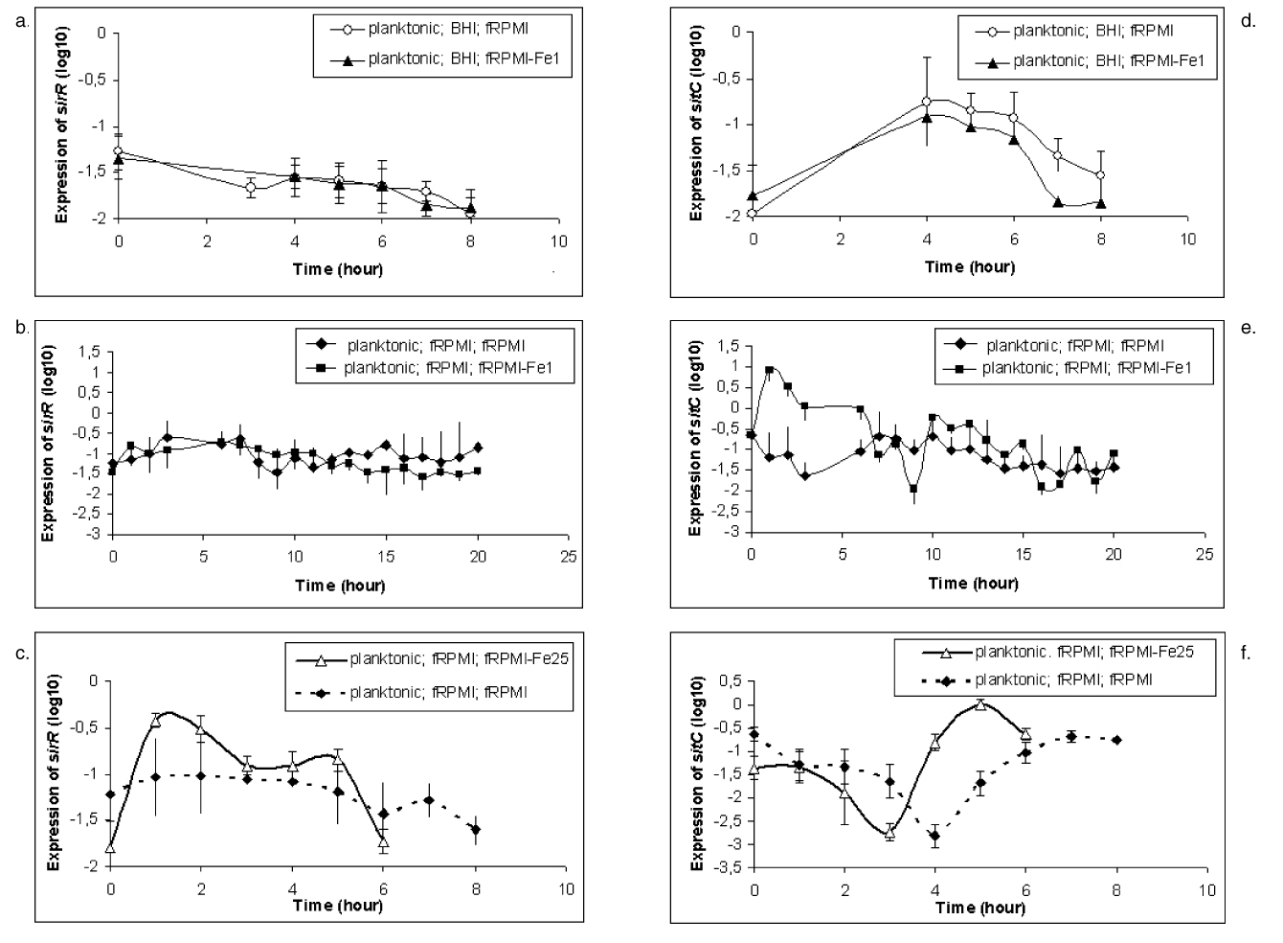
*In vitro *gene expression of *sirR *(a, b and c) and *sitC *(d, e and f) in planktonic bacteria. Gene expression is quantified as log10 (cDNA/gDNA) in the y-axis. The line that links the data points helps to clarify the results at each time point measured. The error bars represent standard deviations. Fifteen samples from three independent cultures were assessed at each time point. Time is given in hours in the x-axis. (a) and (d): bacteria pre-incubated overnight in BHI and incubated in fRPMI (full line with empty circle) or fRPMI-Fe1 (full line with filled triangle). (b) and (e): bacteria pre-incubated overnight in fRPMI and incubated in fRPMI (full line with filled diamond) and in fRPMI-Fe1 (full line with filled square). (c) and (f): bacteria pre-incubated overnight in fRPMI and incubated in fRPMI (dotted line with filled diamond) or in fRPMI-Fe25 (full line with empty triangle).

After overnight pre-incubation of *S. epidermidis *in BHI and resuspension of the bacteria in fRPMI-Fe1 and in fRPMI (Fig. 2a) the expression of *sirR *remained at a constant low level, independently of the Fe-content of the medium. The same results were obtained when fRPMI was used for overnight pre-incubation instead of BHI (Fig. 2b). However, after pre-incubation in fRPMI and re-incubation in fRPMI versus fRPMI with 25 μM FeCl_3 _(fRPMI-Fe25), differences in *sirR *expression were observed (Fig. 2c). In fRPMI-Fe25 *sirR *expression increased during the first 2 hrs (one-way ANOVA; Bonferroni; p < 0.001), followed by a decrease (one-way ANOVA; Bonferroni; p < 0.05) to the level of expression of *sirR *observed in fRPMI.

After 4 hrs and for all subsequent time points, a similar expression of *sirR *was found irrespective of pre-incubation and incubation conditions (Fig. 2a and 2c).

The expression of *sitC *was considered representative for the expression of the *sitABC *operon. Expression of *sitC *was examined in function of the time and Fe-content of pre-incubation and incubation media. For bacteria that were pre-incubated in BHI and re-incubated in fRPMI-Fe1 and in fRPMI, the expression of *sitC *was not significantly different (two-way ANOVA) (Fig. 2d). Pre-incubation of *S. epidermidis *in fRPMI, followed by resuspension in fRPMI and in fRPMI-Fe1, showed that the expression of *sitC *was significantly (two-way ANOVA; Bonferroni; p < 0.05) different between the Fe-containing and the Fe-free environment during the initial part of the growth curve (Fig. 2e). After t = 6 hrs, expression of *sitC *was identical in both media (two-way ANOVA). The expression of *sitC *significantly decreased from t = 0 until t = 20 hrs both for bacteria in Fe-rich and Fe-limited medium (one-way ANOVA; Bonferroni; p < 0.05).

For bacteria pre-incubated in fRPMI and incubated in fRPMI versus fRPMI-Fe25, significant differences in the expression of *sitC *were observed (Fig. 2f). From t = 4 hrs onwards, the expression of *sitC *in bacteria incubated in fRPMI-Fe25 was more pronounced than in bacteria incubated in fRPMI (two-way ANOVA; Bonferroni; p < 0,001). In addition, bacteria pre-incubated in fRPMI had a significantly higher initial expression of *sitC *than bacteria pre-incubated in BHI (two-way ANOVA; Bonferroni; p < 0.001).

### Effect of Fe on growth of sessile bacteria

**Figure 3:**
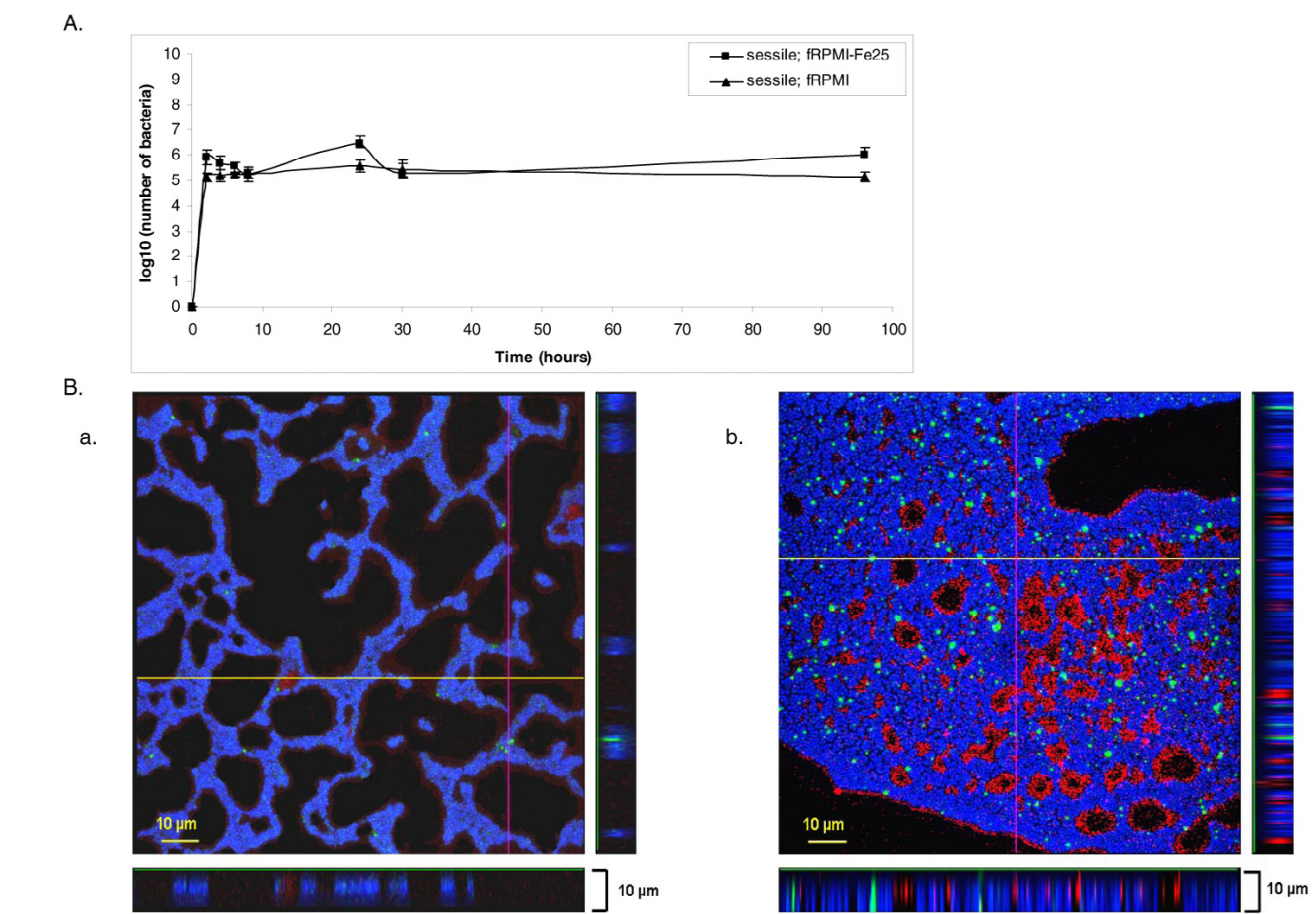
A. The number of bacteria is quantified via the log10 of the number of copies of *gmk*. Bacteria were pre-incubated overnight in fRPMI and incubated together with catheter fragments in fRPMI (filled diamonds) or in fRPMI-Fe25 (filled squares). The error bars represent the standard deviations. The line that links the data points helps to clarify the results at each time point measured. The time is given in hours in the x-axis. Fifteen samples from three independent cultures were assessed at each time point. B. Confocal laser scanning micrographs of sessile bacteria Bacteria were grown overnight in fRPMI and re-incubated together with polyurethane coated glass fragments in fRPMI-Fe25. Pictures were taken after 4 hrs (a) and after 24 hrs (b) of incubation. For each time point images show the xy-, xz- and yz-planes. The xy and orthogonal images of *Staphylococcus epidermidis *biofilms showed living cells (blue, syto9 stained) embedded in extracellular matrix (red, Wheat germ-agglutinin Alexa fluor^® ^633 stained) whereas the green areas (sytox orange strained) represent the dead bacteria. Experiments were performed in triplicate and biofilms were viewed at 100 × magnification.

After overnight pre-incubation of bacteria in fRPMI, bacteria were incubated in fRPMI or fRPMI-Fe25 with added catheters, and real-time quantitative PCR of the *gmk *housekeeping gene was used to determine bacterial load (Fig. 3A). In fRPMI as well as in fRPMI-Fe25 from 2 hrs till 8 hrs of incubation, approximately 10^5 ^bacteria per catheter were measured. After one day and four days of incubation, more sessile bacteria were detected in fRPMI-Fe25 than in fRPMI (two-way ANOVA; Bonferroni; p < 0.05). Similar results were obtained after sonication and plating of the catheters (data not shown).

During these *in vitro *experiments approximately 10^7^–10^8 ^planktonic bacteria per ml sample were measured after 2 hrs and 10^8 ^bacteria per ml sample after 6 hrs in fRPMI; in fRPMI-Fe25, 10^7^–10^8 ^bacteria per ml were measured after 2 hrs and 10^9 ^after 6 hrs (data not shown).

Confocal laser scanning microscopical data confirm the results obtained with gDNA quantification. After 4 hrs of incubation in fRPMI (data not shown) and fRPMI-Fe25 (Fig. 3B) clusters of cells and PIA production could be visualised. Preliminary data indicate higher production of extracellular matrix in fRPMI than in fRPMI-Fe25 after 4 hrs (data not shown). The thickness of the biofilm was up to 10 μm in some places. After 1 day of incubation in fRPMI-Fe25 multilayered clusters of bacteria with extracellular matrix production could be visualized. The overall thickness of the biofilm was 10 μm. Limited numbers of dead cells were scattered throughout the biofilm. In fRPMI fewer bacteria were visible although multilayered bacterial clusters of approximately 10 μm with extracellular matrix could also be visualized (data not shown). Preliminary data indicate that after one day more extracellular matrix is present in fRPMI than in fRPMI-Fe25 (data not shown). Our data also indicate that a one-day old biofilm is not substantially different from a four-day old biofilm (data not shown).

### Expression of *sirR *and *sitC *in sessile versus planktonic bacteria *in vitro*

Differences in expression of *sirR *between planktonic and sessile bacteria were determined after overnight pre-incubation of bacteria in fRPMI and incubation in fRPMI or fRPMI-Fe25 with added catheters.

**Figure 4:**
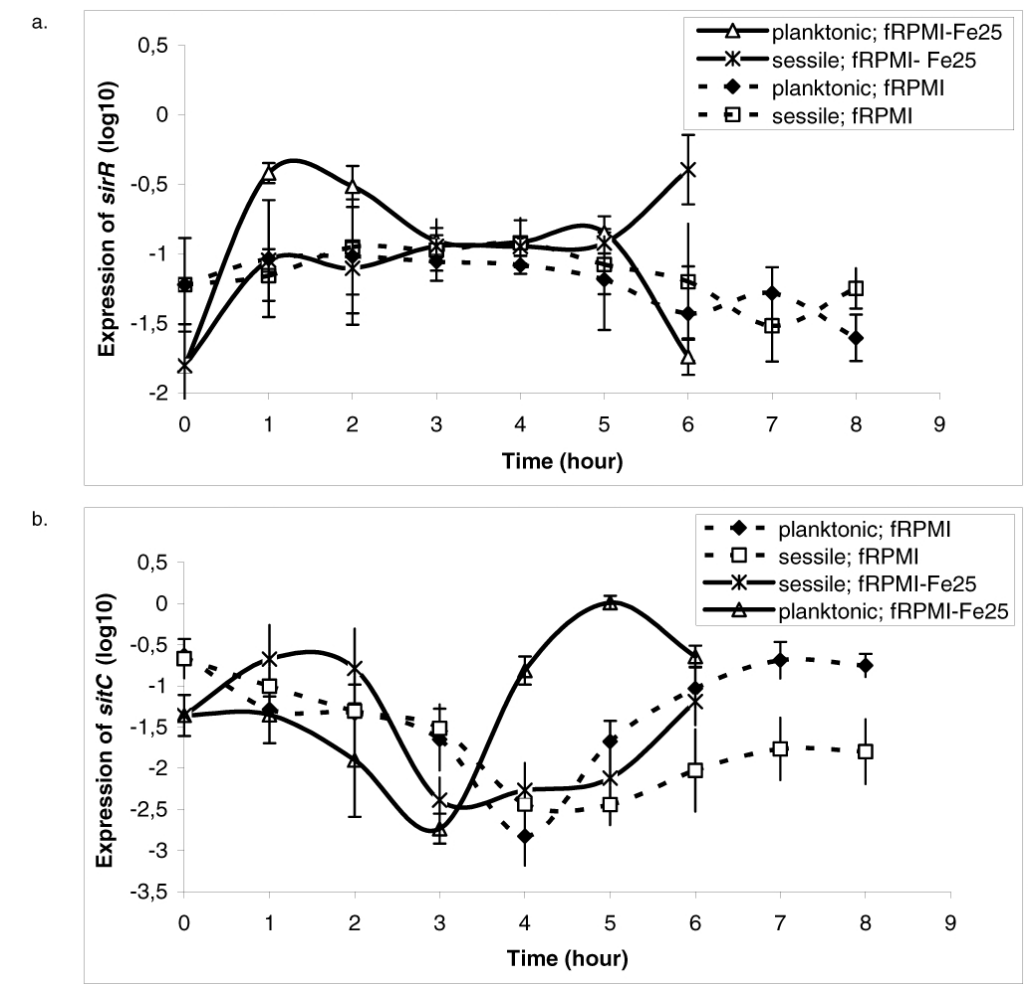
*In vitro *gene expression of *sirR *(a) and *sitC *(b) in sessile versus planktonic bacteria. Gene expression is quantified as log10 (cDNA/gDNA) in the y-axis. Time is given in hours in the x-axis. The line that links the data points helps to clarify the results at each time point measured. The error bars represent standard deviations. Fifteen samples from three independent cultures were assessed at each time point. Bacteria were pre-incubated overnight in fRPMI and incubated together with catheter fragments in fRPMI (sessile: dotted line with empty squares; planktonic: dotted line with filled diamonds) or in fRPMI-Fe25 (sessile: full line with crosses; planktonic: full line with empty triangles).

Initially, a significantly increased expression of *sirR *was observed both in planktonic and sessile bacteria in fRPMI-Fe25 (Fig. 4a). This increase was more pronounced in planktonic than in sessile bacteria (two-way ANOVA; Bonferroni; p < 0.01). After this initial phase, expression was similar, independent of the Fe concentration or growth mode of the bacteria. At t = 6 hrs the expression of *sirR *in sessile bacteria in fRPMI-Fe25 increased significantly (two-way ANOVA; Bonferroni; p < 0.001) in contrast to the expression of *sirR *in planktonic bacteria in the same medium which decreased (one-way ANOVA; Bonferroni; p < 0.001).

Comparison of the expression of *sirR *in sessile bacteria after pre-incubation in BHI versus pre-incubation in fRPMI and incubation in fRPMI and in fRPMI-Fe25 did not show significant differences (data not shown).

The expression of *sitC *in sessile versus planktonic bacteria was analysed in bacteria that were pre-incubated overnight in fRPMI and subsequently incubated in fRPMI or fRPMI-Fe25 with catheters (Fig. 4b). In fRPMI, the expression of *sitC *in planktonic bacteria (one-way ANOVA; Bonferroni; p < 0.001) and sessile bacteria (one-way ANOVA; Bonferroni; p < 0.01) was significantly down regulated during the first 4 hrs. After that, *sitC *expression was up regulated in planktonic bacteria (one-way ANOVA; Bonferroni; p < 0.05) but not in sessile bacteria. After 5 hrs in fRPMI the expression of *sitC *in planktonic bacteria was significantly higher than in sessile bacteria (two-way ANOVA; Bonferroni; t = 5 hrs: p < 0.05 and t > 5 hrs: p < 0.001).

In fRPMI-Fe25, the expression of *sitC *for planktonic bacteria was initially down regulated until t = 3 hrs (one-way ANOVA; Bonferroni; p < 0.05) followed by a significant increase in expression until t = 5 hrs (one-way ANOVA; Bonferroni; p < 0.001) (Fig. 4b). For sessile bacteria in fRPMI-Fe25, the expression of *sitC *remained constant from incubation until t = 2 hrs. After a decrease in expression of *sitC*, from t = 2 hrs until t = 3 hrs (one-way ANOVA; Bonferroni; p < 0.001), expression slowly but significantly increased at t = 6 hrs (one-way ANOVA; Bonferroni; p < 0.001). The expression of *sitC *differed significantly between sessile and planktonic bacteria in this Fe-rich medium from t = 4 hrs onwards (two-way ANOVA; Bonferroni; p < 0.001). The up-regulation of *sitC *expression as well for sessile as for planktonic bacteria started sooner in fRPMI-Fe25 than in fRPMI. The expression of *sitC *for sessile bacteria in fRPMI-Fe25 at t = 6 hrs was also significantly higher (two-way ANOVA; Bonferroni; p < 0.01) than the expression in fRPMI.

The expression of *sitC *in sessile bacteria after pre-incubation in BHI and incubation in fRPMI with and without Fe after 6 hrs, was the same as observed in sessile bacteria after pre-incubation in fRPMI and incubation in fRPMI with and without Fe (data not shown).

**Figure 5:**
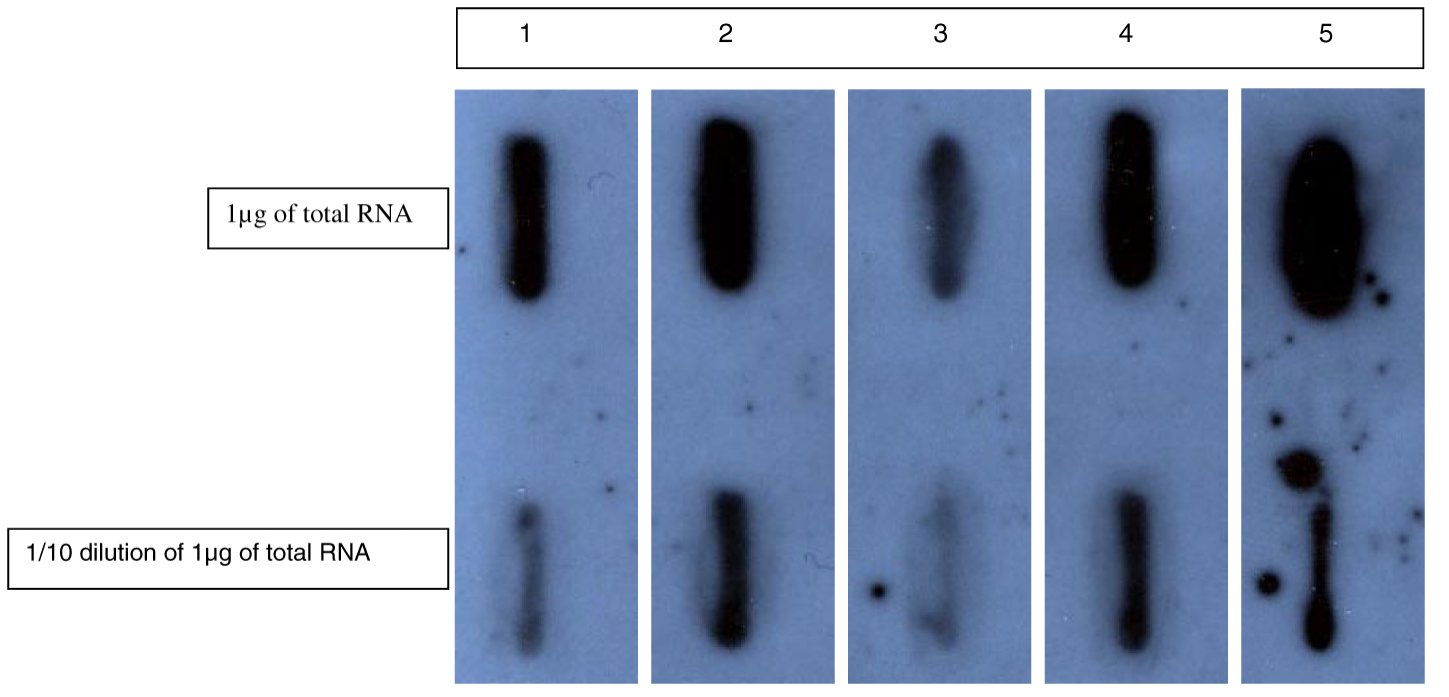
RNA dot blot. RNA samples were taken from bacteria pre-incubated overnight in fRPMI and incubated together with catheter fragments in fRPMI-Fe25. In the first row, each blot contains 1 μg of total RNA, in the second row each blot contains a 1/10 dilution of 1 μg of total RNA. The probe used for the hybridization is the *sitC*-specific RNA probe. Lane 1: RNA from planktonic bacteria after overnight incubation in fRPMI; Lane 2: RNA from sessile bacteria grown in fRPMI-Fe25; t = 2 hrs; Lane 3: RNA from planktonic bacteria grown in fRPMI-Fe25; t = 2 hrs; Lane 4: RNA from sessile bacteria grown in fRPMI-Fe25; t = 6 hrs; Lane 5: RNA from planktonic bacteria grown in fRPMI-Fe25; t = 6 hrs;

To obtain confirmation of these results, total RNA extracts were analyzed through RNA blotting with a DIG labelled *sitC*-specific RNA probe. After overnight pre-incubation in fRPMI and incubation with catheter fragments in fRPMI-Fe25, sessile and planktonic samples were randomly chosen at t = 0 min, t = 2 hrs and t = 6 hrs. Similar results were obtained in comparison to the real-time quantitative PCR (Fig. 5).

### The expression of *sirR *and the *sitABC operon in vivo*

**Figure 6:**
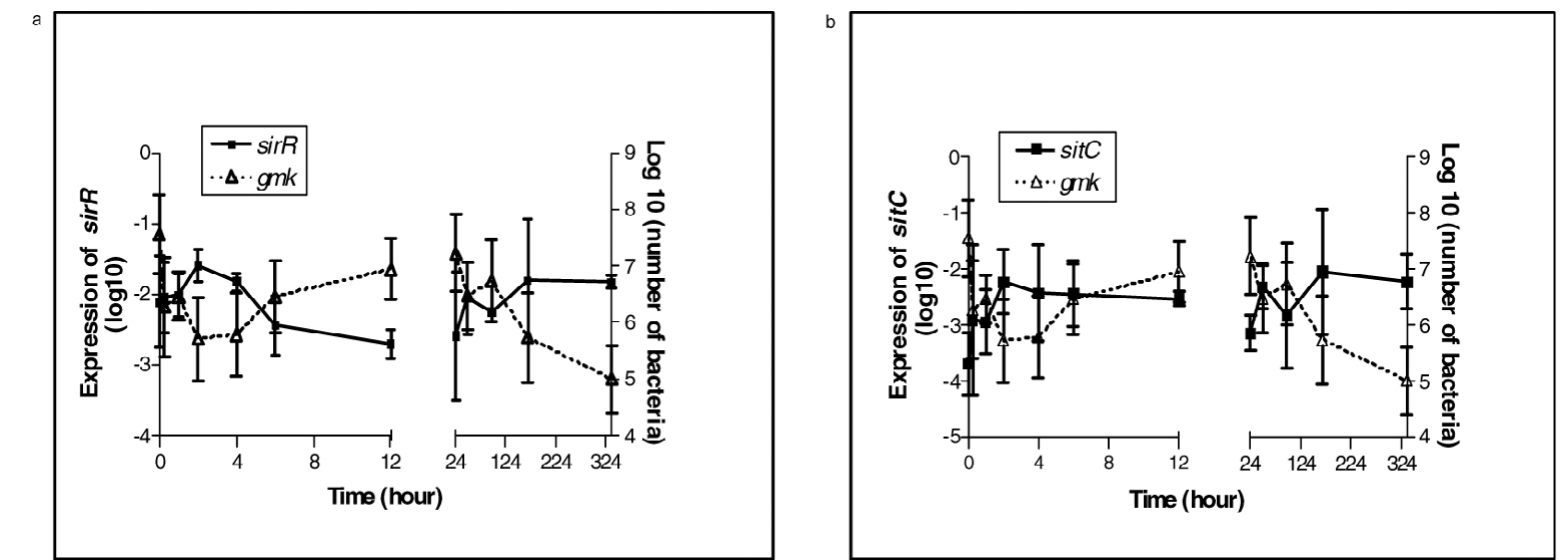
*In vivo *gene expression in sessile bacteria from implantation up to 2 weeks after implantation. Expression levels at time 0 represent the expression levels on the catheter just before implantation. In the left y-axis, gene expression (full line with filled square) is quantified as the log10 (cDNA/gDNA). The right y-axis gives the evolution of the number of sessile bacteria during *in vivo *foreign body infection. The number of bacteria is quantified by the log10 of the number of copies of *gmk *(dotted line with unfilled triangles). The error bars represent the standard deviations. The line that links the data points helps to clarify the results at each time point measured. The time is given in hours in the x-axis. Sixteen independent samples were assessed at each time point.

*In vivo*, the expression of *sirR *in sessile bacteria increased immediately after implantation followed by a rapid decrease from 2 hrs until 12 hrs after implantation. The expression of *sirR *varied 0.5 log between t = 0 hr and t = 2 hrs (one-way ANOVA; Bonferroni; p < 0.05) and 1.1 log between t = 2 hrs and t = 12 hrs (one-way ANOVA; Bonferroni; p < 0.01). The expression levels at t = 12 hrs and t = 24 hrs were significantly lower than the expression levels at later time points (one-way ANOVA; Bonferroni; p < 0.05). After 48 hrs the expression stayed at a constant level (Fig. 6a).

*In vivo*, the expression of *sitC *in sessile bacteria increased significantly (one-way ANOVA; Bonferroni; p < 0.01) from t = 0 hr to t = 2 hrs. It increased 1.4 log between t = 0 hr and t = 2 hrs and 0.9 log between t = 2 hrs and t = 24 hrs; the expression level at t = 0 hr was significantly lower than the expression level at all subsequent time points (one-way ANOVA; Bonferroni; p < 0.05) (Fig. 6b).

*In vivo*, the expression of *sitA *and *sitB *was similar to the expression of *sitC *in sessile bacteria (data not shown).

The amount of bacteria on the catheter during the two weeks incubation period was quantified via the number of copies of genomic DNA (Fig. 6). During the two weeks implantation period, the number of bacteria decreased from 4.10^7 ^bacteria per catheter at implantation to 1.10^5 ^bacteria per catheter at explantation two weeks later (one-way ANOVA; Bonferroni; p < 0.05) (Fig. 6)

## Discussion

The impact of Fe on Fe-regulated genes is difficult to assess because of our incomplete understanding of Fe uptake. Although several studies have addressed Fe uptake by staphylococci, substantial gaps in our understanding of this process remain. An additional difficulty in CoNS is the existence of differences in siderophore production between different strains. Lindsay & Riley [19] reported Fe-regulated production of siderophores in 31% of 39 *S. epidermidis *isolates and constitutive production of siderophores in most other isolates of *S. epidermidis *tested. Some isolates apparently had no detectable siderophore production under the test conditions used. The genetic background of these differences in siderophore production and the possible link to virulence are not yet established. To determine the nature of siderophore production in the strain used in this study, we investigated growth and siderophore production in Fe-depleted and Fe-replete conditions. From these data it appears that siderophore production is present and is Fe-regulated in the strain 10b that we used in our experiments. Further studies on biofilm associated strains with constitutive siderophore production are clearly needed. For this study we only used *S. epidermidis *10b because this strain was isolated from a well defined case of catheter infection and has a 100% infection rate in our *in vivo *foreign body infection model.

In planktonic bacteria in media without Fe we found that siderophore production started only after 6 hrs of growth. This could be due to the limited sensitivity of the assay, implying a very low siderophore production by the bacteria during the first 6 hrs of incubation [8]. Alternatively and according to Somerville et al [20], Fe acquisition in *S. aureus *mostly occurred between post-exponential and stationary phase. Extrapolation of these data to our findings for *S. epidermidis *could explain the lack of siderophore production in fRPMI during the first 6 hrs of incubation. Later studies on *S. epidermidis *however suggested TCA cycle activity, necessitating increased Fe import, in the early exponential growth phase of *S. epidermidis *[21]. The amount of siderophore production in planktonic bacteria at a free Fe concentration of 1 μM was very low. In fRPMI supplemented with transferrin bound Fe, a significantly higher siderophore production was found than in a medium with a comparable concentration of free Fe (data not shown). Although siderophores have a higher affinity for Fe than transferrin [7], Fe-bound transferrin is less accessible to bacteria than free Fe.

The size of the initial inoculum may affect the impact of Fe on bacterial growth. For high inocula (10^7 ^cells/ml or more) the effects of absence or presence of Fe were less pronounced. In this study the lowest inoculum used was – due to technical reasons – 10^6 ^cells/ml. Consistent with the findings of Matinaho *et al*. [3] we found that an increase of the free Fe concentration up to 1–2 μM effectively improved bacterial yield. Higher concentrations of Fe starting from 1 μM FeCl_3 _had little additional effect on bacterial yield (data not shown).

We examined both *in vivo *and *in vitro*, the expression of the *sitABC *operon and *sirR *over time and the effect of Fe on the expression of these genes in *S. epidermidis *FBI. We have previously shown that our methodology is sufficiently sensitive to document the time-dependent induction *in vitro *and *in vivo *of genes involved in biofilm matrix formation such as *icaA *and *icaC *which are present in the *S. epidermidis *strain 10b [22]. In *in vitro *cultures, biofilm formation could also be observed via confocal laser scanning microscopy. These data confirmed that after 1–2 hrs *in vitro*, the first bacterial cells were seen to attach to the surface of the catheter and after 4–6 hrs in fRPMI, with or without Fe, an extracellular matrix was formed. After 1 day *in vitro*, multiple dense layers of bacterial cells embedded in an extracellular matrix were observed.

Different pre-incubation conditions (BHI; Fe-replete and fRPMI) were used to differentiate between bacteria with and without induced siderophore production. According to Ahn *et al*. [23] BHI contains approximately 7,8 μM free Fe although the Fe content may differ between different lots of BHI. Incubation was in fRPMI with defined Fe concentrations. In our sessile versus planktonic *in vitro *experiments, using inocula of 10^7^–10^8 ^cells/ml, the post-exponential phase starts round 4 hrs and 6 hrs for fRPMI with Fe and fRPMI respectively. Taking into account that siderophore expression in response to Fe-depletion takes a few hours to reach maximal levels, we can assume that gene expression patterns in bacteria for the first few hours after re-incubation reflect differences in intracellular Fe concentrations. A rapid increase in intracellular Fe concentrations might be expected after induction of siderophores due to pre-incubation in a Fe-depleted medium and incubation in Fe-replete media. No increase can be expected after pre-incubation in fRPMI and incubation in fRPMI. Pre-incubation in BHI will lead to minimal siderophore induction and a slower response to Fe-depleted growth conditions upon incubation

In planktonic bacteria pre-incubated in BHI, a low *sirR *expression was mirrored by a gradually up-regulated expression of *sitC*, independently of the Fe concentration. The level of expression of *sitC *was higher after overnight pre-incubation in fRMPI compared to bacteria grown in BHI. Somewhat surprisingly, incubation in 1 μM FeCl_3 _gave an initial and transiently higher *sitC *expression than in fRPMI. Other studies using Northern blots [5,9] found no expression of *sitC *in Fe-replete medium but they studied expression after 18 hrs of growth. To validate the results from our *in vitro *tests, total RNA extracts from planktonic and sessile bacteria grown in fRPMI-Fe25 were also analyzed through RNA blotting with a DIG labelled *sitC*-specific RNA probe. Expression of *sitC *was observed on the RNA blot for sessile and planktonic bacteria after 2 and 6 hrs of growth in a Fe repleted medium (25 μM FeCl_3_).

From these data it appears that expression of *sirR *in planktonic bacteria is transiently increased when high siderophore expression and extracellular Fe concentration coexist, probably resulting in high uptake of extracellular Fe. Our data are also consistent with the regulatory role of SirR in the expression of the *sitABC *operon in planktonic bacteria. Significant variability between this study and previous studies [5,9] regarding gene expression of *sirR *in planktonic bacteria could be due to the transient nature of the gene expression. This suggests that single point measurements of gene expression might not be adequate to study the interactions between genes and the impact of environmental conditions. The increase in *sitC *expression in the exponential/post-exponential phase in fRPMI-Fe25 and fRPMI could be due to the need for Fe and for Fe-acquisition.

Several reports indicate that the Fe concentration also affects biofilm formation [3,21]. It has been shown [24] that for *Pseudomonas aeruginosa*, the concentration of Fe is critical for the shift from a sessile to a planktonic growth mode. According to Lyte *et al*. [25], biofilm formation of *S. epidermidis *is stimulated by a sufficient acquisition of Fe. In sessile bacteria, for most genes observed so far, there is a clear and lasting down-regulation of expression compared to the same genes in planktonic bacteria consistent with the low metabolic activity in biofilm-associated bacteria [26,27]. Analysis of the expression patterns of *sirR *and *sitC in vitro *showed not the usual down-regulation in sessile bacteria compared to their expressions in planktonic bacteria.

Our findings indicate that the expression of *sirR *in sessile bacteria is less affected by the pre-incubation conditions and the Fe content of the incubation medium than in planktonic bacteria.

The inverse relation between expression of *sirR *and *sitC *observed in planktonic bacteria was less pronounced in sessile bacteria *in vitro*. This suggests that other factors might affect *sitC *expression in addition to *sirR *or that the interaction between the *sitABC*-operon and *sirR *is different in sessile bacteria. According to Hill *et al*. [5], it is possible that SirR functions as a Mn^2+ ^rather than a Fe^2+^-dependent repressor. They showed that the transcriptional regulator MntR in *S. aureus *is responsive to either Mn^2+ ^or Fe^2+ ^levels [17]. Further studies will be needed to establish the role of Mn^2+ ^on *sirR *and *sitABC *in FBI.

According to Weinberg [18] sessile bacteria need a much narrower Fe concentration range to form a biofilm than planktonic bacteria need during their growth. This implies that the regulatory mechanisms leading to biofilm formation have to be more stringent than in planktonic growth. Thus the high expression of *sirR *in the early phase of biofilm formation could reflect a more efficient regulation of several secondary metabolism associated components. Studies have also suggested that higher concentrations of Fe are needed for attachment [25,28] and for the high metabolic activity in the initial phase of biofilm formation [26]. In a recent study of Fe involvement in biofilm formation by *P.aeruginosa*, it was found that these bacteria need active Fe transport to acquire sufficient intracellular Fe for biofilm development [29]. Deighton & Borland [30] and Vuong *et al*. [21] on the other hand have shown that biofilm formation by *S. epidermidis *is enhanced in a Fe depleted environment. Quantification of sessile bacteria via real-time quantitative PCR of the *gmk *gene showed that in the *in vitro *experiments, the amount of sessile bacteria during the first hours of incubation was higher in fRPMI-Fe25 than in fRPMI. However, after approximately 6 hrs of incubation, the amount of sessile bacteria was the same for both media. The transiently higher expression of *sitC *in the early phases of biofilm formation in fRPMI-Fe25 and the hypothesis that the *sitABC *operon could be a Fe transporter are consistent with an increased growth rate. In fRPMI-Fe25, the decrease in number of bacteria from t = 2 hrs until t = 8 hrs coincides with a decrease in *sitC *expression and was followed by an increase in cell numbers until t = 24 hrs and a second decrease after t = 30 hrs. In fRPMI on the other hand, the number of bacteria increased till t = 2 hrs and stayed almost constant over the rest of the observation period. In addition, these data are not necessarily in contrast with earlier studies [21,30] that measured biofilm formation through production of extracellular polysaccharides irrespective of bacterial counts and showed that low Fe concentration stimulates extracellular polysaccharide production in exponential as well as stationary phase bacteria [30]

The *in vivo *situation is different from the *in vitro *situation because most of the Fe is transferrin bound [28]. In human serum, the total amount of available Fe (free Fe plus glycoprotein-bound Fe) is between 9 μM and 31 μM with free Fe at approximately 10^-18 ^M [31]. Preliminary *in vitro *data showed differences in the expression of *sirR *and *sitABC *in sessile and planktonic bacteria in a medium with transferrin bound Fe compared to a medium with free Fe or without Fe (data not shown). Briefly, for planktonic bacteria *sirR *expression stayed constant over a period of 8 hrs in a medium with transferrin bound Fe. Meanwhile *sitC *expression was initially high in comparison to Fe limited medium or medium with free Fe and its expression decreased later on. In sessile bacteria *sirR *expression decreased after 3 hrs of incubation in a medium with transferrin bound Fe in contrast to its expression in a medium without Fe or with free Fe. In our rat model, *sirR *expression decreased also after 12 hrs and 24 hrs of incubation in comparison to its expression at other *in vivo *time points. *SitC *expression was less dependent on the Fe content (free Fe, transferrin bound Fe or Fe limited).

In our *in vivo *rat model, the amount of the bacteria decreased slightly between implantation and explantation two weeks later, because of detachment of the bacteria from the catheter. The *gmk *gene was used for bacterial quantification of sessile bacteria during our *in vivo *and *in vitro *studies. This was in all phases of the biofilm shown to be the most accurate method [32]

During gene expression studies, the transient expression peak after 2 hrs of implantation for all genes could be due to the adaptation to the new environment. The expression of *sitABC *was not inversely related to the expression level of *sirR*. High expression of s*irR *in itself may not necessarily lead to inhibition of the *sitABC *operon unless SirR is complexed with Fe^2+^. A higher intracellular concentration of *sirR *might however allow a more stringent and rapid response to changes in the intracellular Fe concentration. It remains to be determined how the expression of *sirR *is up regulated in sessile bacteria.

## Conclusion

We conclude that in planktonic bacteria, expression of *sirR *is inversely correlated with *sitC *expression. In sessile bacteria our data suggest that the link between *sirR *and *sitC *expression is less stringent. In planktonic bacteria, *sirR *expression depends on the incubation conditions. In sessile bacteria, the expression of *sirR *and *sitC *is elevated in the initial phase of biofilm formation and after a transient decrease remains constant independently of the Fe content of the medium.

The different expression patterns of *sirR *and *sitC *in sessile versus planktonic bacteria warrant caution in the extrapolation of data obtained in planktonic bacteria to sessile bacteria.

## Methods

### Bacterial strains and growth conditions

A previously well-characterized *Staphylococcus epidermidis *strain (strain 10b) was used [33,34]. This strain was isolated from a patient with a proven catheter related bloodstream infection.

For culture, Brain Heart Infusion, (BHI-Oxoid) was used unless otherwise specified.

For experiments with defined Fe concentrations, RPMI 1640 medium (Sigma-Aldrich) was used. RPMI 1640 was depleted of Fe (fRPMI) as previously described [5] with some modifications. 50 mM Hepes buffer (Sigma-Aldrich) was added and Fe was removed by overnight batch incubation with 6% Chelex 100 (Sigma-Aldrich) at room temperature. Afterwards 0.07 mmol CaCl_2 _(Sigma-Aldrich, ultrapure), 0.7 mmol MgSO_4 _(Sigma-Aldrich, pro-analyse) and 0.3 g glutamine were added to one liter of RPMI 1640 and the pH was adjusted to a range between 7.2 and 7.4. The mixture was filter sterilized. The theoretical calculated concentration of Fe in the medium after addition of MgSO_4_, CaCl_2 _and HCl is negligible (approximately 9,39 nM).

Fe-rich medium was obtained by addition of 1 μM FeCl_3 _or 25 μM FeCl_3 _(Sigma-Aldrich).

### Growth in different media

To determine variation in gene expression between planktonic and sessile bacteria, bacteria were grown overnight in BHI or in fRPMI.

Twenty μl of a frozen bacterial stock culture was inoculated into 5 ml BHI (grown for 14 hrs) or inoculated in 5 ml fRPMI (grown for 18 hrs) in a shaking incubator at 37°C [32]. After centrifugation for 5 minutes at 3020 × g (RC 5B Plus, Sorvall), the pellet was resuspended in fRPMI or fRPMI with Fe until an OD_600 _of approximately 0.5 was reached and catheter fragments of approximately 7 mm were added to the medium.

To establish a growth curve for planktonic bacteria, 40 μl frozen stock culture was inoculated in 10 ml fRPMI and grown overnight for 18 hrs in a shaking incubator at 37°C. After 18 hrs, this bacterial suspension was resuspended in 100 ml fresh fRPMI or fRPMI-Fe1 until an OD of approximately 0.005 was reached. A sample was taken every hour from 0 until 9 hrs, from 14 hrs until 20 hrs, after 25 hrs and after 30 hrs. The absorbance of the samples was measured at a wavelength of 600 nm.

### Gene identification, cloning and quantification of copy number

The sequences of the genes of interest were recovered from the complete genome of the non- biofilm forming *S. epidermidis *strain ATCC 12228 (*sirR*: EMBL X99128; *gmk*: guanylate kinase: EMBL AF270133 plus others; *sitABC*: NC_002976).

PCR was performed on a GeneAmp PCR System 9700 (PE Applied Biosystems). All primers were provided by Eurogentec (Table 1). The PCR-products of all genes were cloned in the pGEM-T easy vector system (Promega) according to the instructions of the manufacturer and resulting in pGEMsirR, pGEMsitA, pGEMsitB and pGEMsitC. Pure plasmid DNA was obtained using the High Pure Plasmid Isolation Kit (Roche Diagnostics). The fragments obtained after amplification with the primers mentioned above were 542 bp long for the *sirR *gene, 580 bp for *sitC*, 540 bp for *sitB *and 546 bp for *sitA*. Preparation of the isolates and sequence analysis through automated capillary electrophoresis on the ABI 310 (PE Applied Biosystems) were performed as described by De Baere *et al*. [35]. Assembly and editing of sequencing products was done with Chromas version 1.45 (School of Health Science Griffith university, Southport Queensland, Australia) and ClustalW [36]. The obtained sequence was compared to known sequences in the genbank by use of Blast 2.0 [37] and ClustalW.

Gene quantification was performed with the Genequant RNA/DNA calculator (Amersham Pharmacia Biotech) at a wavelength of 260 nm and a cuvette path length of 10 mm. The number of gene copies per μl plasmid was 1.3 × 10^10 ^for *sirR*, 2.56 × 10^10 ^for *gmk*, 2.6 × 10^10 ^for *sitA*, 1.9 × 10^10 ^for *sitB *and 1.8 × 10^10 ^for *sitC*.

### Detection of siderophore activity

During the growth of the bacteria, the siderophore activity was measured with the Chrome Azurol S Liquid Assay. The assay was performed as described [8]. Briefly, the sample was centrifuged during 5 min at 3020 × g at 4°C (RC 5B Plus, Sorvall). 500 μl supernatant was added to 500 μl of the CAS assay solution. The CAS assay solution contains 2 mM CAS, 1 mM Fe (1 mM FeCl_3_-6H_2_O in 10 mM HCl), piperazin buffer (Sigma-Aldrich Chemie) and hexadecyltrimethylammoniumbromide (HDTMA) (Sigma-Aldrich). The CAS solution contained 0.121 g CAS (Sigma) in 100 ml water. To create the piperazine buffer, 30 ml water was added to 4.307 g piperazine, followed by the addition of 6.75 ml concentrated HCl to obtain an optimal pH of 5.6. Afterwards 10 μl shuttle solution (0.2 M 5-'sulfosalicylic acid) was added to the mixture of supernatant and CAS assay solution. After 15 min incubation at room temperature the absorbance was measured at a wavelength of 630 nm. The absorbance of siderophore units, correlated with the siderophore concentration can be calculated as a percentage of siderophore units: ((*A*_r_-*A*_s_)/*A*_r_) × 100 with *A*_r _the absorbance of the medium with CAS assay solution and shuttle solution at a wavelength of 630 nm and *A*_s _the absorbance of the culture medium with CAS assay solution and the shuttle solution at a wavelength of 630 nm.

### Model for *in vivo *catheter infection

Experiments were performed as described [32] with some modifications concerning the anaesthesia and euthanasia. Catheter fragments, pre-incubated with bacteria were placed on ice and implanted immediately in ex-germ-free Fisher rats. The anaesthesia of rats was induced with urethane and during the procedure the rats were kept anaesthetised with a combination of 20 % urethane and 80% oxygen. After shaving the back of each rat and disinfecting the skin, 8 catheter fragments were inserted subcutaneously in each rat. A total of 200 catheters were implanted in 20 rats and explanted over a period of maximum 2 weeks. For catheter explantation, animals were euthanized with 0.5% CO_2_. The skin was disinfected and catheter segments were gently removed from the subcutaneous tissue. From each rat 8 catheters were used for nucleic acid extraction.

### RNA and genomic DNA isolation and cDNA synthesis

*In vivo *and *in vitro *DNA and RNA-extraction from sessile bacteria and *in vitro *DNA and RNA extraction from planktonic bacteria were performed as described by Vandecasteele *et al*. [32]. Briefly, for bacteria in suspension, a volume of bacterial culture with a maximum of 10^9 ^CFU (colony forming units) was pelleted for 5 minutes at 3020 × g. The pellet was suspended in 500 μl acidified phenol:chloroform 5:1 pH 4.5 (Ambion) at room temperature and added with 500 μl NAES buffer (50 mM NaOAc, pH 5.1; 10 mM EDTA; 1% SDS) to a FastRNA tube-blue (BIO 101). For sessile bacteria, the catheters were removed from the culture or the rat and rinsed with 1 ml medium or 0.9% NaCl respectively. Subsequently catheters were added to FastRNA tube-blue (BIO 101, Carlsbad, California, USA) with acidified phenol:chloroform and NAES buffer. Afterwards the Fastprep™ instrument (FP 120, BIO 101) was used. After separation of the gDNA from the RNA, the remaining 100 μl, containing the RNA, were purified with the RNeasy mini kit (Qiagen), and treated with RNase free Dnase (Qiagen) according to the manufacturers' instructions. Finally, RNA was eluted in 60 μl RNase-free water.

Reverse transcription was performed as described by Vandecasteele *et al*. [32]. Briefly, we used 100 U MMLV reverse transcriptase with the supplied buffer (Promega), 20 U Rnasin (Promega), 100 μM random hexamers (Amersham Pharmacia Biotech), 1 mM of each dNTP, 9 μl RNA for *in vitro *planktonic experiments and 36 μl for catheter experiments in a total volume of 60 μl.

Reaction conditions were as follows: preheating of the RNA sample for 10 min at 72°C, followed by addition of the reaction mix. cDNA was prepared from this mixture through incubation for 1 hr at 42°C followed by heating at 99°C for 2 min for enzyme denaturation and rapid cooling to 4°C.

### Real-time PCR

Amplification and quantification were performed on the ABI Prism 7700 Sequence detection System (PE Applied biosystems). The Taqman primers and probes were designed by Primer 3 [38] and controlled by Primer Express 1.0 (PE Applied Biosystems). Primers and probes are summarized in Table 2.

The actual gene quantification was performed as described by Vandecasteele *et al*. [34]. Briefly, 2 μl gDNA or cDNA, 12.5 μl 2 × Taqman PCR mastermix (PE Applied Biosystems), 0.9 μM of each primer and 0.2 μM probe in a final volume of 25 μl were used for the real-time PCR reaction. Thermal cycling conditions were the following: 2 min at 50°C, 10 min at 95°C followed by 45 cycles of 15 sec at 95°C and 1 min at 60°C.

During each run a standard dilution of the plasmid with known quantity was included to permit gene quantification using the supplied software according to manufacturer's instructions. In this study a relative quantification has been used. The number of copies of cDNA per ml (a measure of the amount of mRNA) was divided by the number of bacteria per ml. This quotient represents the amount of gene expression (expression of RNA) per viable bacterium.

### Quantification of bacteria via real-time quantitative PCR of *gmk *during *in vivo *and *in vitro *infection

In order to evaluate the number of bacterial cells in biofilms, the number of copies of *gmk *genomic DNA recovered from each *in vivo *and *in vitro *catheter segment was used. As previously demonstrated [32], the number of *gmk *gDNA copies per catheter correlates very well with the number of CFU per catheter.

Consistent with the findings of Vandecasteele *et al*. [32] the number of planktonic cells in our *in vitro *experiments was also quantified with the number of copies of *gmk *genomic DNA.

Primers for plasmid construction of the *gmk *gene are given in table 1; primers and probe of *gmk *gene for real-time PCR are given in table 2.

### Statistical methods

Statistical analysis was performed with Prism (Graphpad software).

As described by Vandecasteele *et al *[39], all data were log10 transformed to fulfill the requirements of normality.

For the *in vitro *data, two hypotheses were tested. A significant change in gene expression levels over time within one group (sessile or planktonic) was tested with one-way ANOVA analysis. A significant difference in the evolution over time of the gene expression levels between the sessile versus the planktonic group was tested with two-way ANOVA analysis. When the ANOVA analysis was significant, two-side univariate tests with a correction for multiple comparisons were done (Bonferroni test) to locate the significant differences. For statistical analysis, fifteen samples from three independent cultures (five samples from each culture) were assessed at each time point.

For the *in vivo *data, one-way ANOVA was used. If there was a significant evolution of the expression levels over time, the two-side Bonferroni multiple comparisons method was used to determine which time-points differed at α = 0.05, with a correction for multiple comparisons.

For statistical analysis, 16 independent samples were assessed at each time point *in vivo*.

### RNA slot blot analysis

After 18 hrs of pre-incubation in fRPMI, the culture was incubated in fRPMI-Fe25. At random chosen time points, RNA was extracted and purified as described above. After RNA extraction, quantification of the RNA was performed through spectrophotometry. For slot blot analysis, a dilution series starting from 1 μg total RNA was applied to a positively charged nylon membrane (F. Hoffmann-La Roche). Using the DIG-RNA labelling kit (SP6-T7) (F. Hoffmann-La Roche) a *sitC *specific RNA probe was made starting from pGEMsitC according to manufacturer's instructions. The RNA samples were hybridised overnight at 50°C in Dig Easy hyb (F. Hoffmann-La Roche) with DIG-labeled *sitC*-specific RNA probe. Hybridizing RNA's were visualized using the chemiluminescent substrate CSPD.

### Confocal microscopy: viability and matrix staining

Visualisation of *in vitro *biofilm formation on catheter fragments was performed as described by Pintens *et al*. (submission in progress). Briefly, glass disks (Menzel GmbH, Germany) coated with a polyurethane layer, were used. Preparation of biofilms on the disks is performed in a 24-well plate in pre-incubation and incubation conditions similar to our *in vitro *experiments. After incubation in the bacterial suspension the disks were washed to remove planktonic cells with 1 ml of fresh medium. For matrix visualisation, the disks were incubated in Wheat germ-agglutinin Alexa fluor^® ^633 labeled (Molecular probes Eugene, OR, USA. excitation at 633 nm and emission at 647 nm). After washing with phosphate buffered saline (PBS), the disk was incubated with syto9 (Molecular probes Eugene, OR, USA; excitation at 480 nm and emission at 500 nm) and sytox orange (Molecular probes Eugene, OR, USA; excitation at 547 nm and emission at 570 nm), two components for live-dead staining of the bacteria. After incubation the disks were washed with PBS. On different randomly chosen locations on each surface, micrographs were taken with a LSM 510 confocal laser-scanning microscope (CLSM; Zeiss, Jena, Germany) with an arrangement of filtersets and lasers as described by Pintens *et al*. (submission in progress). Digital image analysis of the CLSM optical thin sections was performed with the Zeiss LSM software (version 4.1).

## List of abbreviations used

Fe, iron; Fe^3+^, ferric iron; CoNS, coagulase negative staphylococci; FBI, foreign body associated infections; Mn, manganese; Fe^2+^, ferrous iron; BHI, brain heart infusion; fRPMI, RPMI 1640 depleted of iron; *A*, Absorbance; OD, optical density; PBS, phosphate buffer saline

## Authors' contributions

JA and EL participated in the RNA analysis and drafted the manuscript. RM and VP participated in the molecular gene expression studies and drafted the manuscript. JVE participated in its design and coordination and helped to draft the manuscript. CM carried out molecular gene expression studies, the dot blot; she did the design and coordination of the study, drafted the manuscript and performed the statistical analysis. All authors read and approved the final version of the manuscript.

## Figures and Tables

**Table 1:** Forward and reverse primers for PCR and sequencing

**Gene**	**forward 5'-...-3'**	**Reverse 5'-...-3'**
***sirR***	GCGACGTGTCCTTTGTATCA	TGCAAAAATATTCATCGCATTT
***sitA***	GACCTAATGGTGCCGGTAAA	TTGGCCCTAAAAACTGCAAC
***sitB***	TTCCTAGCATCTGGCGTCAT	CACGTTAAAGCGCTCCTTTT
***sitC***	CGTGTCGGTGGAAATAAGGT	TGGCTTGTTTCATTTGACCA
***gmk***	GGATAATGAAAAAGGATTGTTAATCG	GCTTCTACGCGCTCTCTTTT

**Table 2:** Primers and probes for real-time PCR

**Gene**	**Forward primer 5'-...-3'**	**probe: labeled FAM-5'and 3'-TAMRA**	**Reverse primer 5'-...-3'**
***gmk***	AAGGTGCTAAGCAAGTAAGAAAGAAATT	ATGCGTTGTTCATATTTTTAGCGCCTCCA	CAACAAGACGTTCTTTCAAGTCATCT
***sirR***	AATATCAAACCACCATCAGTAAGTGAA	AGGGCGTTTAGAAAAGGAAGGCTATGTTGAA	ACGGGCACCTTTATAATGTTTTGT
***sitC***	CATTAACAGATGCTGACGTTGTATTTT	AACTGGAAATGGTTGGTTTGAAAAAGCACTTG	CCTTCCTCACCATTTAAAGTATATTGG
***sitB***	TTTGTCATGTTATTACTCGCACTTGTA	CTGTTGCGAGCATACAAACAGTTGGAATTATCC	AGCTGTAGAAGCTGGAGTGATTAGTAAA
***sitA***	TGCAGAATTAAGTGGTGGACAATT	AACGTGTTCTTGTTGCAAGAGCACTTATGAGTAATAGT	CCAACAAAAGGTTCGTCTAAACAAT
